# Accuracy of periodontal probing depths in the training of undergraduate dental students

**DOI:** 10.1186/s12909-025-08233-0

**Published:** 2025-12-29

**Authors:** Ellen E. Jansen, Andreas Braun, Felix Krause, Patrick Jansen

**Affiliations:** Department for Operative Dentistry, Periodontology and Preventive Dentistry, RWTH Aachen University, Pauwelsstrasse 30, 52074 Aachen, Germany

**Keywords:** Periodontitis, Periodontal probing depths, Dental education

## Abstract

**Background:**

Precise periodontal diagnosis is taught and routinely performed during dental school training. The aims of this study were to determine to what extent the measurement values obtained by students during their clinical training years correspond to those of licensed dentists; to assess the distribution of divergent results relative to measurement positions; and to identify sources of error. The study also investigated whether fewer errors are made in accessible areas such as the anterior teeth and vestibular areas in comparison with inaccessible areas. Finally, it investigated the influence of students’ level of experience on measurement accuracy.

**Materials and methods:**

Data for probing depths in 6858 measurements made by students in comparison with dentists were documented and analyzed statistically. The influence of periodontal pocket depths on measurement accuracy was examined, and it was investigated whether the location of pockets (vestibular/oral, anterior/posterior, quadrant, mesial/medial/distal) influenced measurement divergences.

**Results:**

There were significant differences (*P* < 0.001) between the students’ and dentists’ measurements, which were in agreement in 63% of cases. Differences of 1 mm were observed in 80% of cases, 2 mm in 15.3%, and 3, 4, or 5 mm in fewer than 5%. Measurement differences in the last clinical course were significantly the largest (*P* < 0.01). Accuracy in medial measurement points was significantly better (*P* < 0.001). Differences in measurement accuracy in the anterior and posterior teeth, as well as on the vestibular and oral surfaces, only became apparent after analysis of subgroups for healthy, mild, and severe cases. Patients with severe cases had significantly smaller measurement differences in both the oral and vestibular areas (*P* < 0.02). Healthy patients had significantly better measurement values in the posterior region (*P* = 0.02), and patients with mild conditions had significantly better values in the anterior region (*P* = 0.03).

**Conclusions:**

The results of this retrospective examination show that students already achieve a comparatively high level of measurement accuracy, but that differences occur, particularly in the approximal region. Targeted practical training and structured feedback systems could help further improve diagnostic precision, which is particularly important when there is a recall system with changing practitioners.

## Background

Periodontology is one of the central disciplines in dentistry and is concerned with periodontitis and other inflammatory diseases that affect the periodontium. It therefore plays an important role in tooth preservation, as well as in general treatment planning [11]. Periodontal diseases are among the most common oral diseases worldwide and represent a significant challenge for dental practice, due to their prevalence and their effects on oral and systemic health [23]. Chronic periodontitis in particular, which is characterized by inflammatory destruction of the periodontium, can lead to tooth mobility, tooth loss, and concomitant systemic diseases if left untreated [28]. Timely detection and monitoring of periodontal disease is therefore a decisive factor for successful treatment and long-term tooth preservation [11].

As part of the periodontal diagnostic work-up, assessment of periodontal status is an important component of the clinical findings. This includes assessment of probing depths, clinical attachment level, bleeding on probing (BOP), furcations, mobility, and recessions. The assessment of probing depths plays a central role here. It gauges the clinical pocket depth and provides crucial information about the presence and severity of periodontal disease. It also allows documentation of disease progression and forms the basis for individual treatment decisions [14].

Due to its major clinical relevance, the accuracy of periodontal probing is of particular importance. However, studies show that there is a certain amount of intraindividual and interindividual variability in periodontal probing, which is influenced by various factors. In addition to the level of the examiner’s experience, these include anatomical characteristics, the degree of tissue inflammation, probing pressure, and instrument design [29].

Particularly in the context of dental training, it is therefore essential to teach prospective dentists the importance of precise diagnosis and to provide systematic training in practically implementing it [8]. Dental training faces the challenge of imparting both theoretical knowledge about periodontal diseases and the practical skills required for reliable diagnosis and treatment of them. Numerous studies have shown that targeted training and practical exercises can improve the accuracy of periodontal probing and reduce measurement variability [1, 24]. Incorporating practical diagnostic exercises and structured feedback systems into training curricula is therefore an important part of dental education [8].

In addition to the teaching of basic diagnostic techniques, important aspects of training include making trainees aware of potential sources of error and capable of critically evaluating their own findings. Due to limited visibility and complex anatomical conditions, greater measurement differences can be observed on molars in particular in comparison with the anterior teeth [4, 12]. This problem underlines the need for intensive practical training in all areas of the oral cavity so as to ensure the greatest possible measurement accuracy and prevent misdiagnosis [9].

Periodontal diagnosis is also becoming increasingly important in modern dentistry, as periodontal diseases not only have local consequences for oral health, but are also associated with systemic diseases. Numerous studies have shown associations between periodontitis and cardiovascular diseases, diabetes mellitus, and pregnancy complications [13, 18]. Against this background, precise periodontal diagnosis is becoming an important aspect of interdisciplinary patient care. Dentists have a key role to play here, as the early detection of periodontal diseases is essential for successful treatment and avoidance of systemic complications [20, 21].

In view of this, it is of particular interest to evaluate the accuracy of students’ periodontal probing measurements. The question arises of the extent to which students develop sufficient measurement precision during the course of their training, and in which areas there is still a need for optimization. Earlier studies have shown that students’ measurement accuracy is variable in comparison with that of licensed dentists, but that it can be significantly improved through targeted training measures [7, 27]. The present retrospective evaluation of the data makes it possible to further optimize dental teaching and develop targeted training content aimed at improving diagnostic skills.

The aim of this study was therefore to identify the accuracy of students’ measurements of periodontal probing depths and potential differences in the precision of their findings in comparison with those of licensed dental educators, as part of the current teaching approach used in the Department of Operative Dentistry, Periodontology and Preventive Dentistry, RWTH Aachen University, Germany. Particular attention was given to distinguishing tooth positions as anterior versus posterior, as well as measurement sites at each tooth: mesio-oral, mid-oral, and disto-oral (and corresponding vestibular points). Furthermore, the frequency and extent of measurement discrepancies were analyzed for each localization. The influence of the students’ training level was also analyzed. The results are intended to help further target and develop dental training in the field of periodontal diagnosis and to identify potential weaknesses in practical implementation.

The null hypotheses here were that there are minor deviations in the anterior and vestibular region, greater deviations in the posterior and oral region, and that there is a significant difference between dentists and students that declines as the level of training increases.

## Materials and methods

In this retrospective data evaluation, periodontal diagnostic records for 98 patients in the Department of Operative Dentistry, Periodontology, and Preventive Dentistry at Aachen University Hospital were analyzed. A retrospective study design was chosen to utilize existing clinical records and a large number of measurements, enabling a comprehensive analysis of student and licensed dental educator probing accuracy while capturing the current status quo of teaching to identify areas for improvement and adapt the curriculum accordingly. An additional advantage of this approach was that the measurements were unbiased, as students were unaware of the study and therefore did not alter their performance. Data collection took place between January 2021 and April 2024. Each patient was measured by a single student. Different students were involved over the study period, representing multiple student cohorts. A total of 13,530 periodontal probing depths in these patients, who were regular patients treated within the clinical student course, had been noted by dental students as part of clinical examinations and had been checked and documented accordingly by registered dentists. This study included students in their clinical study period, from the fourth year of study (first clinical course, integrating the fields of cariology, periodontology, endodontology, restorative and pediatric dentistry [ICC1], seventh semester), the fifth year of study (fourth clinical course, integrating the fields of cariology, periodontology, endodontology, restorative and pediatric dentistry [ICC4], tenth semester), and the state examination (SE, the last examination before licensing).

The study was conducted in accordance with the Declaration of Helsinki [30] and was approved by the local ethics committee (CTC-A-Nr.25–261, EK 25–233, RWTH Aachen University). As this retrospective study used fully anonymized routine data, the need for individual informed consent was deemed unnecessary according to German national regulations (§15 of the professional code for physicians in North Rhine and the EU General Data Protection Regulation).

### Periodontal training concept

**Fig. 1:**
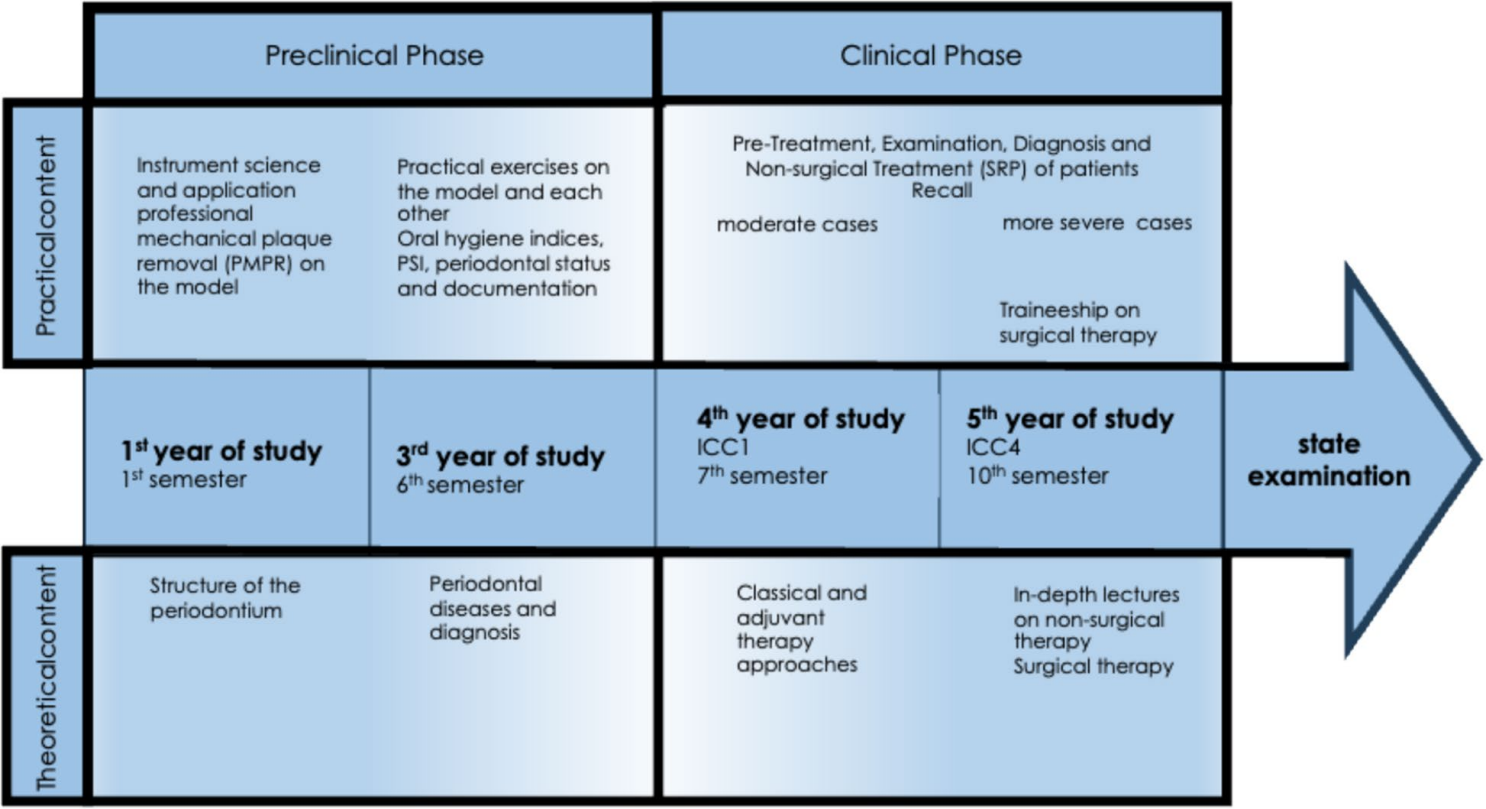
The Aachen Periodontal Therapy Concept (APTC): the structure of the periodontal curriculum in the Department for Operative Dentistry, Periodontology, and Preventive Dentistry at RWTH Aachen University Hospital

Periodontal education at the Department for Operative Dentistry, Periodontology, and Preventive Dentistry at the Faculty of Medicine at RWTH Aachen University is meticulously structured according to a curriculum and is delivered throughout each academic year or semester. Instruction in the Aachen Periodontal Therapy Concept (APTC) follows a clear, consecutive sequence (Fig. 1). The curriculum is structured so that students progress to the next course only after successfully completing the previous one, ensuring a gradual accumulation of clinical experience.

In the first year of study (first semester), students receive initial theoretical and practical exposure to the specialty of periodontology. This phase equips them with basic knowledge of the anatomy, etiology, pathogenesis, and diagnosis of periodontal diseases, and also provides initial practical experience, such as carrying out professional mechanical plaque removal (PMPR) on a model. Subsequently, in the third year of study (sixth semester), theoretical knowledge is deepened and students engage in periodontal diagnostic procedures on models and peers under supervision. During practical training in this phase, which also encompasses oral hygiene instructions and PMPR as well as scaling and root planing (SRP) on the model, specific emphasis is given to the meticulous collection of oral hygiene indices, the periodontal screening index (PSI), and periodontal status. The findings obtained are then documented in a fully digital working environment using a software program (ParoStatus.de). These preclinical practical exercises, both on models and on the students themselves, and the supervision, correction, and feedback provided by the supervising dentists, provide calibration before the clinical study phase. Furthermore, before entering the clinical courses, students are required to complete and pass a pre-clinical state examination, ensuring they have acquired the necessary foundational knowledge and skills to safely participate in patient care.

The skills acquired up to this point are then further refined during the clinical student courses. From the fourth year of study (seventh semester onward), clinical training is integrated into treatment modules within the student courses. In addition to lectures and seminars on classical and adjuvant periodontal therapy approaches, practical exercises on patients take place in the fourth year of study (first clinical course, integrating the fields of cariology, periodontology, endodontology, restorative dentistry, and pediatric dentistry [ICC1], seventh semester). Students pretreat, examine, diagnose, and treat patients with periodontal disease (primarily mild cases). This includes, above all, the assessment of periodontal probing depths as part of periodontal charting and nonsurgical periodontal treatment (SRP). This theoretical and practical knowledge is built on in the fifth year of study (fourth clinical course, integrating the fields of cariology, periodontology, endodontology, restorative, and pediatric dentistry [ICC4], tenth semester). In addition to in-depth lectures on nonsurgical periodontal therapy, there are also seminars and clinical traineeships on surgical periodontal therapy approaches. In the practical exercises, the above approach is applied with a focus on more severe periodontal cases. Between the start of the fourth year of study (ICC1, seventh semester) and the end of the fifth year of study (ICC4, tenth semester), one year of intensive prosthetic training takes place in the Department of Prosthetic Dentistry, Implantology and Biomaterials at RWTH Aachen University Hospital. The practical periodontal components are progressively enhanced throughout the conservative clinical courses. In both the ICC1 and ICC4 courses, each step is supervised, checked, and corrected by licensed dentists who provide constructive feedback, especially in the event of divergences. In this way, a feedback loop is established that supports the students in their learning progress and thus further deepens the theoretical and practical course content that is successively built up (as a spiral curriculum). As part of the state examination (the final examination before licensing), students carry out a periodontal recall, including periodontal charting and scaling and root planing of diseased teeth. This is of course also checked by licensed dentists.

### Data selection

The inclusion criteria for the evaluation were complete periodontal charts with at least 40 measurement sites, documented probing depths at six measurement sites per tooth, and traceable assignment of the examiner (student and dentist). The measurements were always carried out on the same day. On the basis of these criteria, 5505 measurements had to be excluded because not enough measurement points could be collected or the measurements were not carried out on the same day. A total of 49 patient records were included in the study, comprising 6858 measurement points. Of these, 3036 measurements were obtained during the ICC1 course, 2340 measurements during the ICC4 course, and 1482 measurements during the state examination. The study population consisted of 20 women (40.8%) and 29 men (59.2%), with a mean age of 60.9 years (SD = 13.0; median = 61.0; range: 24.1–82.1 years).

### Data collection

**Fig. 2:**
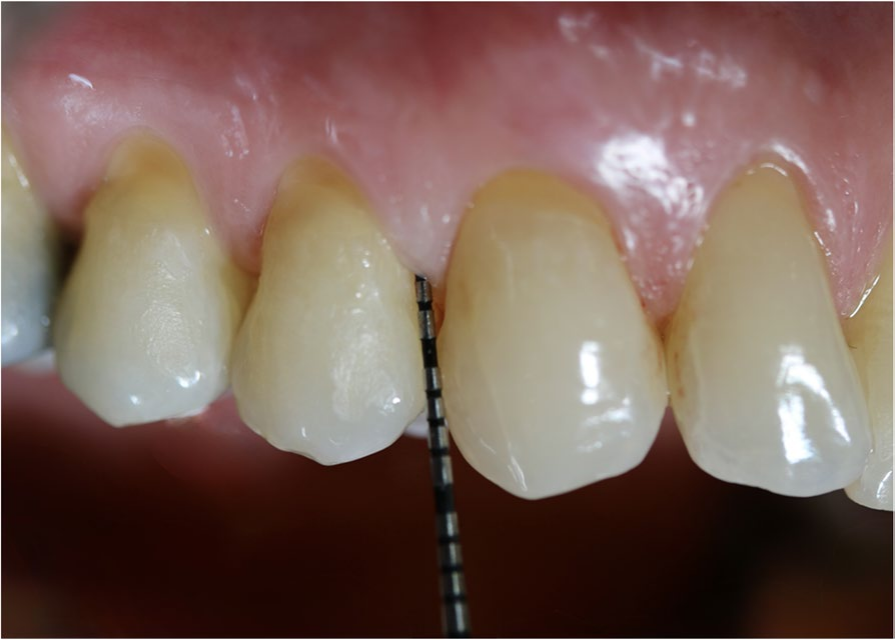
Example measurement of a periodontal pocket with a UNC15 probe in a patient

The standard procedure for measuring periodontal probing depths used a UNC15 probe at the usual six measurement points per tooth (mesio-buccal, mid-buccal, disto-buccal, mesio-oral, mid-oral, disto-oral). Only probing pocket depth (PPD) was analyzed in this study as the focus was on the accuracy of students’ periodontal probing measurements. The distance from the free gingiva edge to the bottom of the pocket was measured, the probe was always inserted perpendicular to the tooth axis with a probing pressure of approximately 0.25 N [5]. Although some patients had gingival recessions, only PPD was assessed, so the presence of recessions did not affect the measurements. The probing depths were documented in millimeters, it was always rounded to the nearest millimeter. Figure 2 shows an example measurement on a patient. The measurements made by the licensed dentists were used as reference values and compared with the students’ findings. All licensed dental educators are clinical experts in periodontology; most have completed the Master’s program in Periodontology at the International Academy of RWTH Aachen. They also participate regularly in internal training as well as continuing education programs to ensure the consistent use of standardized measurement procedures. In the context of dental education, it is essential that all instructors work in a calibrated manner, which is maintained through regular collaboration and supervision within the teaching team, also through senior doctors. Through this continuous teaching experience and practical standardization, the reference measurements were reliable and consistent. Therefore, the absence of a formal pre-study calibration is unlikely to have affected the validity of the results. The students receive calibration in that they measure probing depths in the preclinical courses on calibrated models and on each other (including checks by the supervising dentist).

### Data analysis

For the analysis, all values measured by the students were compared internally (with comparison of the different study years) and with the corresponding measurements by the dentists. The number of identical measurements and the extent of the divergences (in millimeters) were recorded. Deviations of up to 1 mm were categorized as clinically acceptable, while differences of ≥ 2 mm were graded as relevant or significant. The choice of a 1 mm threshold for agreement in periodontal measurements is based on established standards in clinical research. Studies such as those by Badersten et al. [4] and Fitzgerald et al. support this threshold as a workable limit for assessing measurement accuracy [4, 10]. In addition, separate evaluations were carried out for the anterior and posterior teeth, by quadrant, by vestibular or oral location, and by the position of the measurement point on the tooth. These evaluations were carried out for the entire group as well as for the three individual groups — healthy cases (sulcus depths < 3 mm), mild cases (pocket depths 3–5 mm), and severe cases (pocket depths > 5 mm) — to identify potential differences in measurement accuracy depending on the location.

The anonymized data were transferred to an Excel spreadsheet (Microsoft Excel, version 2405) and statistically analyzed using IBM SPSS Statistics for Windows (version 29). For each measurement site, the difference between the two time points was calculated, and these values were then aggregated at the group level (patient health status, student experience level). Consequently, the statistical analysis was based on aggregated group-level differences rather than on individual sites, thereby reducing the impact of within-patient dependencies.

Descriptive statistics were calculated, and Wilcoxon and chi-square tests were performed to identify significant differences between the measurements made by students and dentists. The significance level was set at 0.05.

## Results

**Fig. 3:**
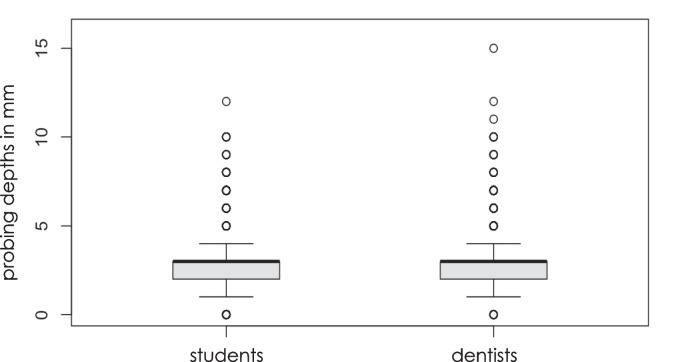
Box plot diagram showing periodontal measurements by students and dentists. The diagram displays the median, quartiles, and potential outliers to demonstrate potential variations in periodontal assessments between the two groups

Data for periodontal probing values at a total of 6858 measurement points, which were recorded by both experienced dentists and dental students in a clinical setting, were retrospectively analyzed. The two groups had a similar main distribution of the probe values, with median and lower and upper quartiles in the range of 2–3 mm (Fig. 3). The completely overlapping boxes indicate comparable central tendencies and dispersion within the interquartile ranges.

However, the extreme values differed significantly: while individual outliers among the students ranged up to a maximum value of 12 mm, outliers up to a value of 16 mm occurred among the course-supervising dentists, influencing the overall impression of the distribution.

**Fig. 4:**
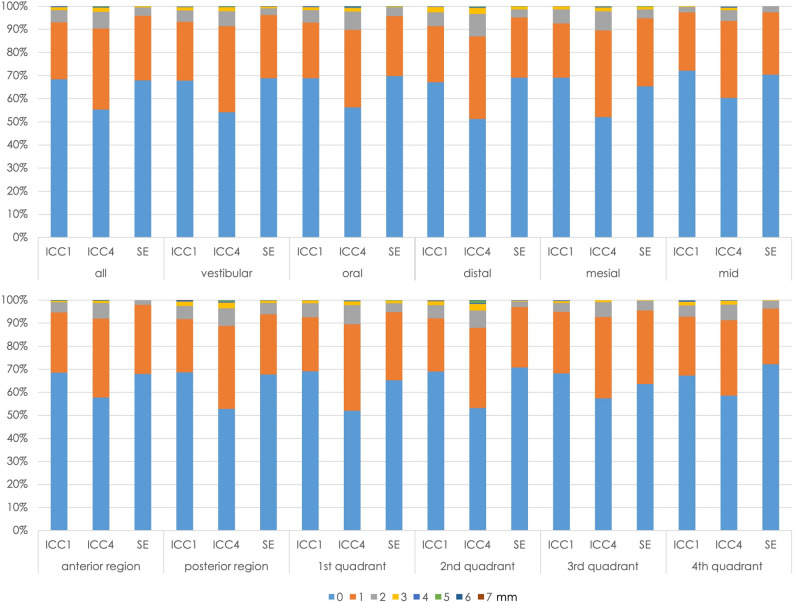
Percentage distributions of measurement differences, analyzed relative to students’ level of knowledge (ICC1 course, ICC4 course, state examination) and by location

The first question is the extent to which the students’ level of experience affected the measurement accuracy. The percentage distribution of the measurement differences was therefore analyzed using a chi-square test in the different periods of study: ICC1, ICC4, and state examination; the data are presented in Fig. 4. Measurement accuracy during the ICC4 course differed significantly from the other two periods (*P* < 0.01). In ICC1 and ICC4, there were also significant differences in the accuracy of the vestibular and oral measurement points (*P* < 0.007). In all three groups, the measurement points (distal, mesial, and medial) differed significantly in the distribution of the divergences (*P* < 0.001). Medial was significantly better in all of the courses (*P* < 0.001), with a difference of 0 mm being the most common and 2–7 mm the least common.

**Fig. 5:**
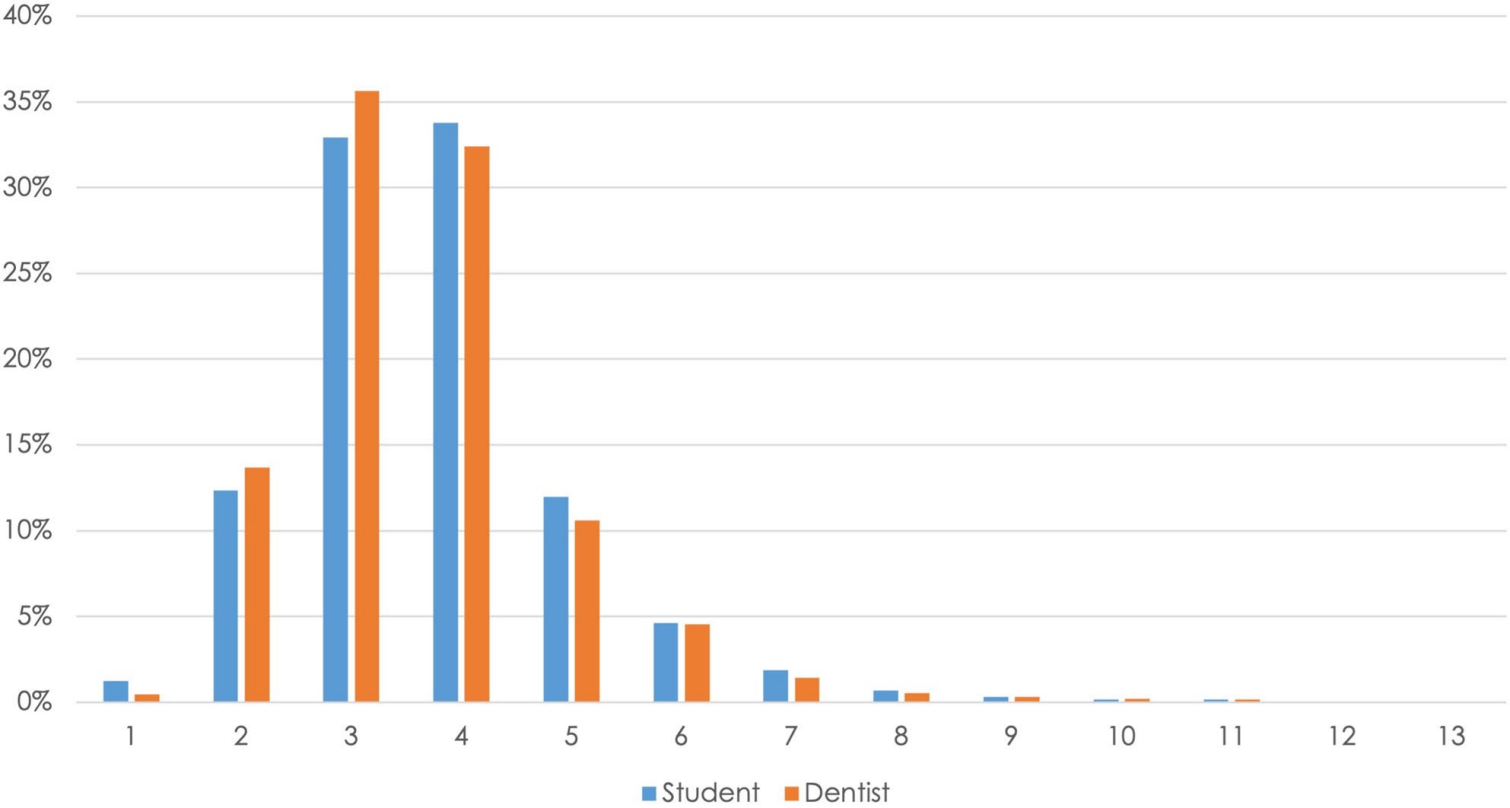
Percentage distribution of the measured probing depths for the dentist and student groups

Secondly, the distribution of the raw data was broken down into how often which probing depths were detected in the two groups (students and dentists). The percentage distribution of the numerical values measured by students and dentists for the probing depths was basically similar in the two groups. There were clear maximum values of over 30% at 3 mm (students 33.8%, dentists 32.4%) and 4 mm (students 32.9%, dentists 35.6%), and over 10% at 2 mm (students 12.3%, dentists 13.7%) and 5 mm (students 12%, dentists 10.6%) (Fig. 5).

The Wilcoxon test was then used to test the correspondence of the values measured respectively at the corresponding points, to assess the students’ measurement accuracy. Significant measurement differences were only observed at 4% of the measurement points (*P* < 0.05); one point was located in the second quadrant, and three each in the third and fourth quadrants.

**Fig. 6:**
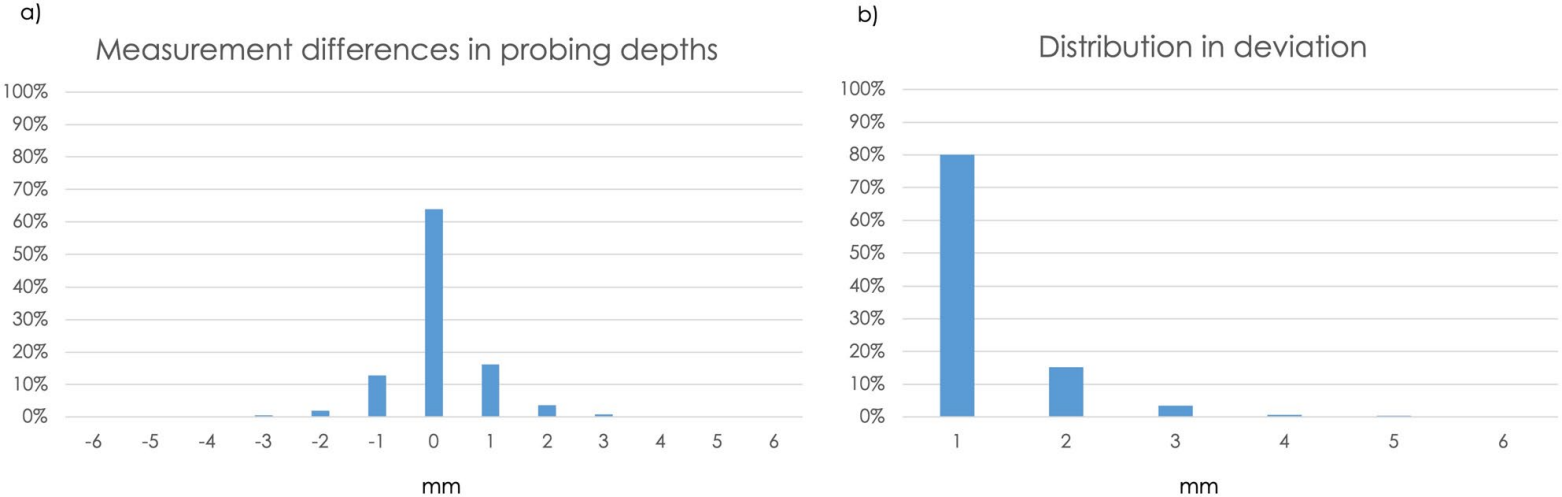
**a** Divergences in measurements of probing depths. **b** Percentage distribution of the amounts of divergence

Subsequently, the differences between the two measured values (dentists and students) were determined for each measurement point. The results showed an even distribution of positive and negative measurement divergences (Fig. 6a). In over 63% of cases, there were no measurement divergences, while in 37% of the measurement deviations there was a divergence of 1 mm in 80% of cases and a divergence of 2 mm in 15.3%. Deviations of 3, 4, and 5 mm occurred in fewer than 5% of cases each (Fig. 6b).

Analysis of patient-related factors revealed no relevant influence of either age or sex on measurement deviation. While a slight increase in deviation with age was suggested (y = 0.004x + 0.2204; R^2^ = 0.0203) and a minimal decrease with male sex (y = −0.0611x + 0.4892; R^2^ = 0.0067) was observed, both effects were negligible.

Basic statistical data were then calculated for the individual subgroups (Table 1). The mean values for the measurement differences and SD underline the previous findings showing that measurement accuracy decreases between ICC1 and ICC4 and then increases again significantly toward the state examination. In addition, the maximum measurement difference decreases with increasing experience level. The severe cases showed the smallest measurement deviations, but also the largest maximum differences.

**Table 1:** Subgroup analysis for the ICC1 and ICC4 courses, state examination, and healthy/mild/severe cases

	**ICC 1**	**ICC 4**	**SE**	**Healthy cases**	**Mild cases**	**Severe cases**
Mean	0.41	0.6	0.36	0.46	0.57	0.38
SD	0.72	0.8	0.61	0.55	0.73	0.72
Median	0	0	0	0	0	0
Minimum	0	0	0	0	0	0
Maximum	7	6	5	2	6	7
IQR	1	1	1	1	1	1
N	3036	2340	1482	312	2610	3936
Significance	A	B	A	C	D	C, D

*IQR* Interquartile range, *SD* Standard deviation, *SE* State examination, *N *Sample size, *ICC1* First Clinical Course, integrating the fields of cariology, periodontology, endodontology, restorative andpediatric dentistry, *ICC4* Fourth Clinical Course, integrating the fields of cariology, periodontology, endodontology, restorative and pediatric dentistry

**Fig. 7:**
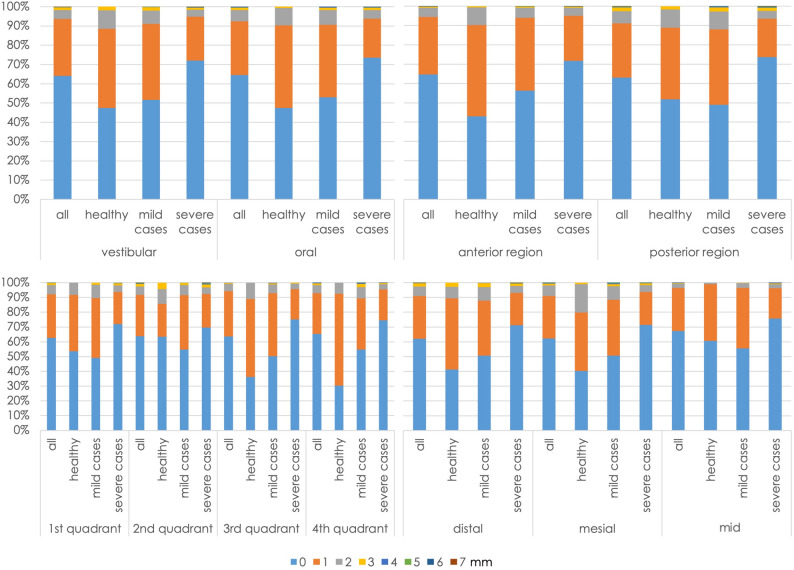
Percentage distributions of measurement differences relative to severity and location

It was also investigated using the chi-square test whether the location or severity of the periodontal disease had any impact on measurement accuracy. There were no significant differences in measurement accuracy between the oral and vestibular measurement sites in any of the three patient groups (healthy, mild cases, severe cases) (*P* = 0.74, 0.79, 0.96, respectively). In both the oral and vestibular cases, severe findings were significantly associated with smaller measurement differences (*P* < 0.02). When the anterior teeth were compared with the posterior teeth, no differences in measurement accuracy were observed for the patients as a whole (*P* = 0.87). In the individual groups, the analysis showed that healthy patients had significantly better measurement values in the posterior region (*P* = 0.02) and patients with mild cases had significantly better values in the anterior region (*P* = 0.03). The medial measurement sites were significantly more precise in all cases than the mesial and distal sites (*P* < 0.01). The severe cases were always measured significantly more accurately at all three measurement points (*P* < 0.01). While no significant differences in measurement accuracy were observed between the four quadrants when looking at all cases, splitting the data into the individual groups again revealed significant differences in measurement accuracy. In all quadrants, the severe cases were again measured best (*P* < 0.001). The full data are shown graphically in Fig. 7.

Another important therapeutic aspect is the extent to which the classifications made on the basis of the measured values correspond. As can be seen from Table 2, patients with mainly slight pocket depths (4–5 mm) were correctly recognized in 68% of cases, while patients with pocket depths of 6 mm or more were correctly diagnosed in 93% of cases. Analyzing the frequency distribution with the chi-square test revealed a highly significant difference between the groups (*P* < 0.0001).

**Table 2:** Percentage concordance of the severity levels diagnosed

		**Dentists’ measurements**	
		**Mild cases**	**Severe cases**
**Students’ measurements**	Mild cases	68.42%	0.00%
	Severe cases	31.58%	92.86%

## Discussion

The present study analyzed the accuracy of measurement of periodontal probing depths recorded by dental students during clinical courses. Measurements made by the dentists supervising the course were used as control values. The aim was to determine possible differences in the precision of the findings and to identify potential sources of error. Significant deviations (*P* < 0.001) were found in the students’ measurements. The measurements were in agreement in 63% of cases and showed a 1-mm difference in 80%, a 2-mm difference in 15.3%, and a 3, 4, or 5-mm difference in fewer than 5%. Significantly larger measurement differences were seen in the ICC4 course (*P* < 0.01). The accuracy of medial measurements was significantly better (*P* < 0.001). Differences in measurement accuracy on the anterior, posterior, vestibular, and oral surfaces became apparent after subgroup analysis. Patients with severe cases had significantly smaller measurement differences in both the oral and vestibular areas (*P* < 0.02). Significantly better values were measured in healthy patients in the posterior region (*P* = 0.02), while mildly affected patients had significantly better values in the anterior region (*P* = 0.03).

These results confirm the findings of earlier studies reporting that periodontal probing is a fundamentally reliable diagnostic method, but one that is influenced by various factors — e.g., the design of the probe and interexaminer variability [15, 22]. Particularly in the follow-up of periodontal diseases, measurement differences can lead to misjudgments regarding the course of disease and thus influence treatment decisions. Incorrect diagnoses of progression may be made or necessary treatment adjustments overlooked. Despite its ease of use in everyday practice, periodontal probing is subject to a certain degree of variability, and individual factors such as probing pressure, access, patient anatomy, and clinical experience can influence the results [25].

This examination of learning progress among students passing from the ICC1 course to the state examination reveals an unexpected pattern: rather than a smooth trajectory, there is a decline during the ICC4 course, followed by a marked improvement at the state examination. This phenomenon can be attributed to the dental training approach used during the clinical study phase at Aachen University Hospital, as mentioned above. During the prior preclinical semester in conservative dentistry (the third year of study), the students complete a comprehensive range of theoretical and practical exercises in periodontology, both on models and on each other. As a result, the students appear to be well prepared for the ICC1 course and thus the start of the clinical study phase, which also has practical conservative and periodontal content. However, the subsequent two clinical courses in the fourth and fifth years of study, which are mainly dedicated to prosthodontics, occur between the two clinical conservative/periodontal courses. Evidently, students enrolled in the ICC4 course have faced significantly fewer periodontological examinations during the previous year. This decline results in a minor reduction in the precision of their practical measurements. However, after a full semester of practical periodontal training, the measurement results show a clear improvement in accuracy at the state examination and a significant improvement in comparison with the previous semester (*P* < 0.01). Other causes could be a possible overestimation of one's own abilities, fatigue during longer clinical sessions or the greater complexity of the patient cases treated in this semester. Cases of overconfidence among senior students are also known from other disciplines. In a study by Costa Filho [6], sixth-year medical students showed greater overconfidence, particularly in difficult dermatological cases [6]. Students in the State Examination (SE) may focus more on the measurements due to the examination situation, which could lead to the best results overall. However, since the study was not designed to directly examine these factors, these remain hypothetical explanations that could be verified in future investigations. The null hypothesis can thus be partially confirmed. A refresher on practical periodontal measurement procedures at the start of the ICC4 course would probably provide a useful aid here.

The facts that most measurements showed no divergences, while again most incorrect measurements indicated a difference of only 1 mm, can be considered acceptable from a clinical point of view, and this is consistent with previous studies on interindividual measurement differences [1, 17]. This underlines the fact that the students already have a largely reliable technique for periodontal probing.

The largest differences were observed at the proximal measuring points. This can be explained by several factors, including limited access, the need for probe angulation and reduced tactile sensitivity. In contrast, mid measurement points are often easier to access, have a straighter tooth surface and allow for more stable guidance of the probe, increasing the reproducibility of measurements. Such challenges at interproximal sites are well documented in the dental literature and are considered a typical limitation of periodontal pocket measurement, especially for inexperienced examiners. Studies show that intraexaminer and interexaminer reproducibility at the mid-buccal sulcus is greater than with interproximal measurements [10]. In contrast to previous studies [16, 19], which have reported greater reproducibility in shallow pockets, the present study found significantly smaller measurement divergences in deep pockets. This seemingly counterintuitive finding among undergraduate students may be explained, on the one hand, by the potentially easier probing of deeper pockets, where following the pocket contour allows a more consistent probe angulation. On the other hand, inflamed tissue may have facilitated probe penetration, making measurements appear more reliable [3]. In addition, a form of confirmation bias may have occurred — when anticipating disease, students may have been more attentive in both probe placement and reading compared to healthy sites.

These findings have important implications for dental training. Although the results by the students in the present study can be rated as good overall, the training content and practical exercises should be focused in particular on probing in the posterior region. Special training programs aimed at correct probing technique, control of probing pressure, and assessment of pocket depths in hard-to-reach areas could help further improve measurement accuracy. Research shows that structured training programs can significantly increase the reliability of periodontal probing [7, 9, 26]. Additionally, it is recommended to refresh practical periodontal measurement procedures at the start of ICC4 to address the potential decline in measurement accuracy that can occur over time or between clinical courses. This refresher session is designed to reinforce the foundational skills in probing technique, including proper angulation, tactile sensitivity, and systematic assessment of pocket depths. It will feature more targeted exercises on applying the optimal probing force, approximately 0.25 N, to ensure consistency and minimize tissue trauma, as well as the use of pressure-calibrated probes, which provide objective feedback and help standardize measurements across students. By combining hands-on practice, immediate feedback from calibrated licensed dental educators, and repeated exercises under controlled conditions, these measures aim not only to refine students’ technique, but also to enhance the reproducibility and reliability of periodontal probing. Ultimately, this structured refresher is intended to reduce discrepancies between student and licensed dental educators measurements, promote confidence in clinical assessments, and ensure a higher overall quality of student performance in periodontal examination. Furthermore, the use of virtual reality (VR) could also be considered. A study by Zhang et al. [31] showed that the combination of a (virtual) jaw model and VR produced good results in periodontal training with regard to supragingival cleaning [31].

However, it is important to acknowledge a key limitation of the study. As with any retrospective study, this investigation is subject to inherent methodological constraints, including the absence of prospective data collection, potential sources of bias, and limited control over confounding factors. The measurements obtained by the dentists, who were designated as the control group, were not verified by the same dentist but by various supervisors of the two courses. In future studies, these should be carried out by a single person in order to exclude interindividual measurement differences within the group. Although the examinations were always carried out by qualified dentists with many years of specialized experience in periodontology, interindividual variations of 1 mm need to be taken into consideration [1, 17]. As this is a retrospective study, pre-study calibration of the dentists was not feasible. However, all participating dentists are closely involved in dental education and regularly conduct periodontal training sessions. This continuous experience and close collaboration help ensure a high degree of standardization and agreement in measurements, allowing them to serve as a practical reference for comparison. Although measurements were performed consecutively by students and licensed dental educators, minor local tissue changes between readings may have slightly contributed to the observed discrepancies.

As it a consequence of the study’s retrospective nature, this aspect cannot be altered. Nevertheless, it is crucial to consider this point when planning future prospective studies. The fact that the study was not conducted in standardized conditions is a limiting factor in the interpretation of the results. Although all of the examiners used the same instruments and the students all underwent repetitive teaching events on the model, peers, and patients as described above, factors such as bleeding, patient movements, and degree of tissue inflammation may influence measurement accuracy.

The use of digital measurement methods or electronic periodontal probes could also help reduce the variability of measurements in the future and thus further standardize the diagnostic work-up [2].

## Conclusion

In summary, this study demonstrates that dental students at Aachen University Hospital generally achieve a high level of accuracy in periodontal probing. However, there is still potential for improvement, particularly at mesial and distal sites and in shallower pockets. These findings emphasize the importance of targeted education and ongoing practical training in periodontal diagnostics to enhance measurement accuracy. Improved accuracy provides a more reliable foundation for treatment planning and follow-up in patients with periodontal disease. Additionally, a structured refresher at the start of ICC4—focusing on probing technique, optimal force application, and standardized calibration—is recommended to maintain and further improve measurement reliability over time.

## Data Availability

The datasets used and/or analysed during the current study are available from the corresponding author on reasonable request.

## Abbreviations

APTC: Aachen Periodontal Therapy Concept
BOP: Bleeding on Probing
ICC1: First Clinical Course, integrating the fields of cariology, periodontology, endodontology, restorative and pediatric dentistry
ICC4: Fourth Clinical Course, integrating the fields of cariology, periodontology, endodontology, restorative and pediatric dentistry
IQR: Interquartile Range
N: Sample Size
PMPR: Professional Mechanical Plaque Removal
PSI: Periodontal Screening Index
SD: Standard Deviation
SE: State Examination
SRP: Scaling and Root Planing
